# CD4/CD8 Ratio Increase in Female Living with HIV Switching to Cabotegravir-Rilpivirine: A Real-Life 24 Weeks Evaluation

**DOI:** 10.3390/pathogens14070633

**Published:** 2025-06-25

**Authors:** Serena Spampinato, Emmanuele Venanzi Rullo, Giuseppe Nicolò Conti, Andrea De Vito, Andrea Marino, Teresa Cirelli, Viviana Coco, Alessia Mirabile, Rossella Fontana del Vecchio, Antonina Franco, Arturo Montineri, Chiara Frasca, Chiara Gullotta, Michele Salvatore Paternò Raddusa, Ylenia Russotto, Aakash Fugooah, Sarah Pulvirenti, Sonia Sofia, Grazia Pantò, Claudia Calì, Roberto Bruno, Eugenia Pistarà, Nunziatina Villari, Carmelo Iacobello, Bruno Cacopardo, Benedetto Maurizio Celesia, Giovanni F. Pellicanò, Francesco P. Antonucci, Giordano Madeddu, Sergio Lo Caputo, Giuseppe Nunnari

**Affiliations:** 1 Unit of Infectious Diseases, ARNAS “Garibaldi Nesima” Hospital, Department of Clinical and Experimental Medicine, University of Catania, 95122 Catania, Italy; dott.ssaserenaspampinato@gmail.com (S.S.); giuseppecontichimica@gmail.com (G.N.C.); andrea.marino@unict.it (A.M.); teresa.cirelli@gmail.com (T.C.); viviana.coco@gmail.com (V.C.); alessia.mirabile@gmail.com (A.M.); chiaragullotta96@gmail.com (C.G.); michelepat93@gmail.com (M.S.P.R.); aakash.fugooah@studenti.unime.it (A.F.); sarah.pulvirenti@libero.it (S.P.); roberto.bruno@unict.it (R.B.); eugeniapistara@gmail.com (E.P.); nancy.villari@gmail.com (N.V.); bruno.cacopardo@unict.it (B.C.); bmcelesia@gmail.com (B.M.C.); giuseppe.nunnari1@unict.it (G.N.); 2 Unit of Infectious Diseases, “G. Martino” University Hospital, Department of Clinical and Experimental Medicine, University of Messina, 98125 Messina, Italy; ylenia.russ@gmail.com (Y.R.); giovanni.pellicano@unime.it (G.F.P.); 3 Unit of Infectious Diseases, Department of Medicine, Surgery and Pharmacy, University of Sassari, 07100 Sassari, Italy; andreadevitoaho@gmail.com (A.D.V.); giordano@uniss.it (G.M.); 4 Unit of Infectious Diseases, Umberto I Hospital, 96100 ASP Siracusa, Italy; r.fontanadelvecchio@gmail.com (R.F.d.V.); antoninafranco18@gmail.com (A.F.); 5 Unit of Infectious Diseases, “G. Rodolico-S. Marco” University Hospital, University of Catania, 95121 Catania, Italy; a.montineri@libero.it (A.M.); chiara.frasca@gmail.com (C.F.); 6 Unit of Infectious Diseases, AOE “Cannizzaro”, 95126 Catania, Italy; sonia.sofia@email.it (S.S.); graziapanto@tiscali.it (G.P.); carmelo.iacobello@gmail.com (C.I.); 7 Unit of Infectious Diseases, ASP Ragusa, 97100 Ragusa, Italy; claudia.cali@studenti.unime.it; 8 Unit of Infectious Diseases, Department of Medical and Surgical Sciences, University of Foggia, 71122 Foggia, Italy; antonuccifp@gmail.com (F.P.A.); sergio.locaputo@unifg.it (S.L.C.)

**Keywords:** HIV, long acting, Cabotegravir-Rilpivirine, CD4/CD8 ratio

## Abstract

In 2022, 20 million women globally were living with HIV, yet they remain underrepresented in clinical trials, including those for antiretroviral treatments (ART). This study assesses the safety and efficacy of the long-acting cabotegravir-rilpivirine (CAB-RPV) regimen in a cohort of 54 women living with HIV (WLWH) over 24 weeks. A retrospective cohort study from the Sardinian HIV Network and Sicilian HIV Cohort (SHiNe-SHiC) included WLWH who switched to CAB-RPV. Primary objectives were achieving and maintaining HIV RNA levels <50 copies/mL at 24 weeks. Secondary objectives included treatment safety, durability, and reasons for discontinuation. Data on demographics, viro-immunological markers, lipid profiles, and treatment interruptions were analyzed. Of 54 WLWH, 46 reached 24 weeks. The median age was 50 years. A total of 71.8% transitioned from dolutegravir (DTG) regimens. Virological suppression was 97.8% at baseline and 95.5% at 24 weeks. Significant increases in the CD4/CD8 ratio (*p* = 0.0076) and decreases in serum creatinine levels (*p* = 0.0109) were observed. Cholesterol, triglycerides, ALT, and AST levels remained unchanged. The CAB-RPV regimen demonstrated significant virological and immunological efficacy and safety in women living with HIV over 24 weeks. Notably, the improvement in the CD4/CD8 ratio and the increase in the percentage of women achieving target not detected (TND) status highlight the regimen’s effectiveness. These findings emphasize the importance of gender-focused research in HIV treatment and the need for equitable access to effective treatment options for women, which is crucial for global efforts to eliminate HIV.

## 1. Introduction

Women account for nearly half of the global population living with HIV, with 20 million affected as of 2022 [1,2,3]. Despite this substantial burden, gender disparities persist in both access to care and representation in clinical research. In 2024, the Commission on the Status of Women emphasized the need to address gender-based inequities in the global HIV response, highlighting the disproportionate impact of the epidemic on women and adolescent girls [4]. These disparities are shaped by a complex interplay of social determinants, biological differences, and systemic barriers to healthcare access [4,5,6].

Sex-based differences in pharmacokinetics, adverse drug reactions, and comorbidity profiles are increasingly recognized as critical to optimizing antiretroviral therapy (ART) [7,8,9]. Moreover, women living with HIV face unique comorbidities, including a higher risk of HPV-related cervical cancer and breast cancer, which further complicate disease management [10]. These risks are compounded by persistent gaps in cancer screening and early diagnosis in this population [11]. Non-AIDS-defining cancers, now a leading cause of morbidity and mortality among people living with HIV, require integrated approaches to care that consider both oncological and virological outcomes [10]. However, women remain underrepresented in clinical trials, particularly in studies evaluating newer ART regimens. A 2016 systematic review reported that women comprised only 20% of ART trial participants and even fewer in studies of curative strategies [12].

Recent efforts have begun to address this gap. Our previous work demonstrated the viro-immunological efficacy and safety of bictegravir/emtricitabine/tenofovir alafenamide (B/F/TAF) in a real-world cohort of women living with HIV (WLWH) [13], with additional evidence suggesting improvements in metabolic health and immune response over 96 weeks [14]. Similarly, dolutegravir-based regimens have shown high efficacy, safety, and tolerability in clinical practice across diverse patient populations, reinforcing the value of integrase inhibitor-based therapies in real-world settings [15]. Cardiovascular complications, another critical concern in this population, have also been highlighted in recent focused reviews [16]. While these findings were encouraging, data on long-acting injectable regimens in this population remain limited.

Long-acting cabotegravir and rilpivirine (CAB/RPV LA) represent a promising alternative to daily oral ART, particularly for individuals facing adherence challenges. The safety and virological efficacy of long-acting cabotegravir and rilpivirine (CAB/RPV LA) have been demonstrated in both clinical trials and real-world settings [17,18,19,20,21,22,23,24,25,26,27,28,29,30,31]. A multicenter study confirmed high levels of satisfaction and virological suppression among individuals receiving CAB/RPV LA [32]. However, evidence on its use in women is scarce. Only one real-world study, by Marta Rosas Cancio–Suárez, has reported virological outcomes in women living with HIV [27]. Additionally, a recent study showed improved CD4/CD8 ratios in people living with HIV, but not in women [30]. This study aims to evaluate the virological and immunological outcomes of CAB/RPV LA in WLWH at 24 weeks post-switch, using data from the same SHiNe-SHiC cohort, while expanding the scope to a different therapeutic strategy.

## 2. Materials and Methods

### 2.1. Study Design and Population

This retrospective observational study was conducted using data from the Sardinian HIV Network and the Sicilian HIV Cohort (SHiNe-SHiC), a collaborative research initiative that collects clinical and laboratory data from people living with HIV (PLWH) across multiple centers in Southern Italy. The present analysis focused on women living with HIV (WLWH) who transitioned to long-acting cabotegravir and rilpivirine (CAB/RPV LA) as part of routine clinical care.

The primary endpoint was the proportion of participants achieving virological suppression, defined as HIV RNA <50 copies/mL, at 24 weeks following the switch. Secondary endpoints included treatment safety, durability, and reasons for discontinuation. This study builds upon previous analyses of the SHiNe-SHiC cohort [13], which evaluated outcomes with oral bictegravir-based regimens in a similar population.

### 2.2. Data Collection

Demographic, clinical, and laboratory data were extracted from the SHiNe-SHiC database. Variables included age, duration of HIV infection, comorbidities, prior antiretroviral regimens, and laboratory parameters, such as CD4 count, HIV RNA levels, lipid profile, creatinine, liver enzymes, and glucose. Virological response was categorized into four groups: target not detected (TND), <50 copies/mL, 51–200 copies/mL, and >200 copies/mL.

### 2.3. Statistical Analysis

Descriptive statistics were used to summarize baseline characteristics. Continuous variables were reported as medians with interquartile ranges (IQRs) or means with standard deviations (SDs), depending on distribution. Categorical variables were expressed as frequencies and percentages. Normality was assessed using the Shapiro–Wilk test. Paired comparisons between baseline and follow-up values were performed using the Student’s *t*-test or Wilcoxon signed-rank test, as appropriate. A *p*-value < 0.05 was considered statistically significant. Outliers were identified using the ROUT method with a Q value of 1%. This threshold was selected to minimize the influence of extreme values on statistical significance while also avoiding the exclusion of too many samples, given the already limited cohort size. All statistical analyses were performed using GraphPad Prism 9.5.1 for MacOS (GraphPad Software, Boston, MA, USA, https://www.graphpad.com (accessed on 13 April 2025)).

### 2.4. Ethical Considerations

The study protocol was approved by the Provincial Ethics Committee of Messina (protocol code 34/17, approval date: 22 May 2017) and conducted in accordance with the Declaration of Helsinki. All participants provided written informed consent. Data handling complied with the European Union General Data Protection Regulation (GDPR), and all patient information was anonymized to ensure confidentiality.

## 3. Results

A total of 46 women living with HIV (WLWH) who had completed at least 24 weeks of follow-up after switching to long-acting cabotegravir and rilpivirine (CAB/RPV LA) were included in the analysis, out of 54 eligible participants. The median age was 50 years (interquartile range [IQR]: 36–57), and the majority (92%) were of Caucasian ethnicity. The median duration of HIV infection was 17.5 years (IQR: 6.75–29). The most frequently reported comorbidities were dyslipidemia and hypertension (Table 1). Laboratory parameters are summarized in Table 2.

Regarding prior antiretroviral therapy, most participants (70.4%) transitioned from two-drug dolutegravir-based regimens (2DR), while others had previously received three-drug dolutegravir-based regimens (5.5%), bictegravir-based regimens (13%), or tenofovir alafenamide (TAF)-based regimens (16.6%) (Table 1).

Two participants discontinued CAB/RPV LA during the observation period: one due to virological failure (HIV RNA > 200 copies/mL) and one due to an injection site reaction (ISR).

**Figure 1:**
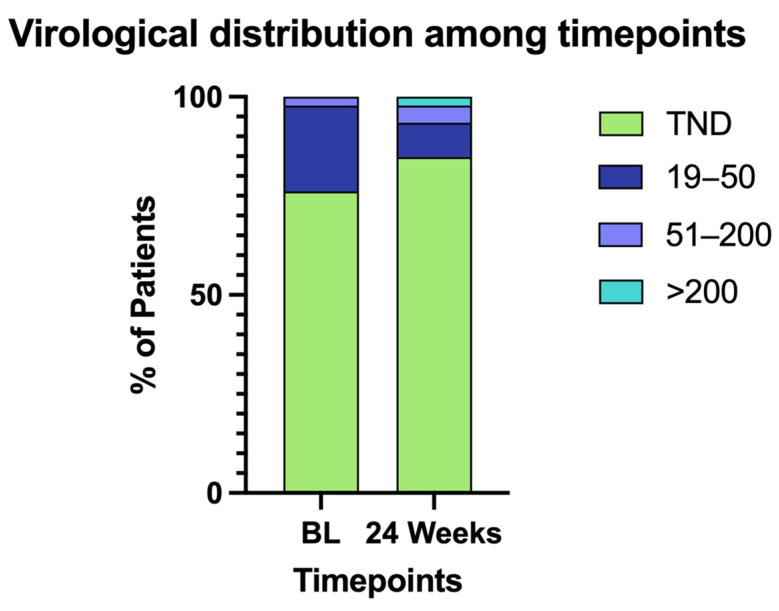
Distribution of patients in virological categories among timepoints.

At baseline, 97.8% of participants had HIV RNA levels <50 copies/mL, with 77.7% achieving “target not detected” (TND) status. At week 24, virological suppression was maintained in 95.5% of participants, and the proportion with TND increased to 86.6% (Figure 1, Table 3 and Table 4).

**Figure 2:**
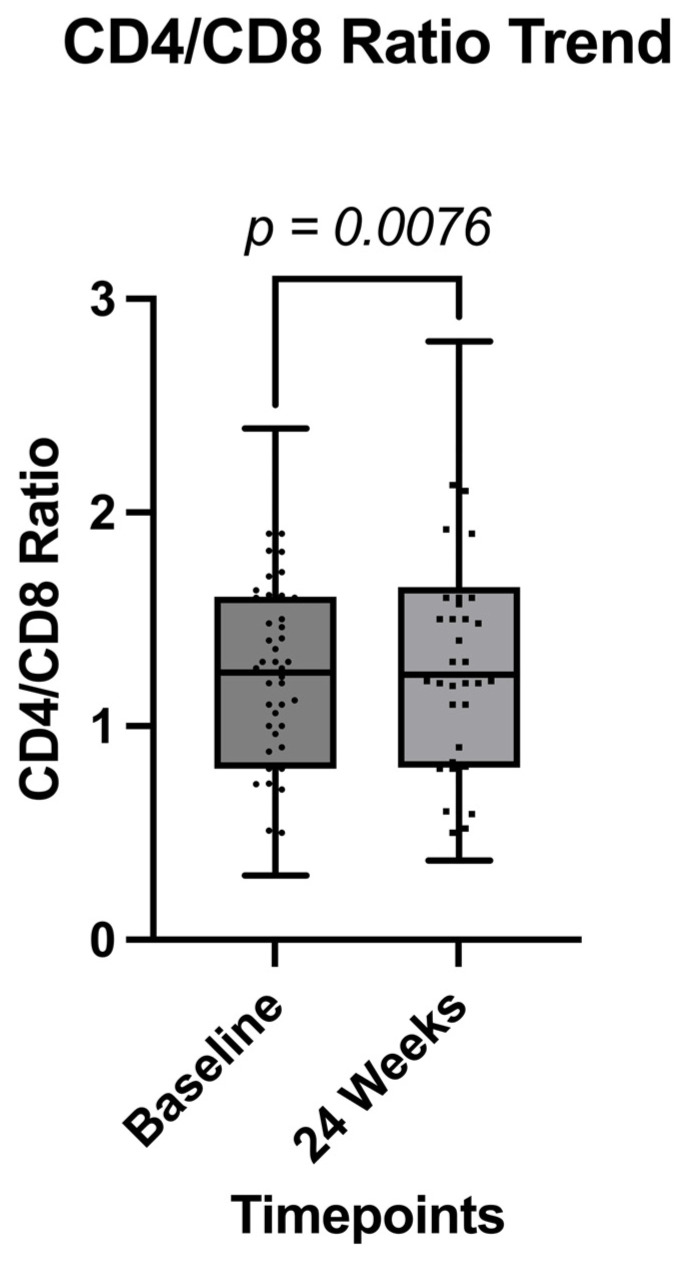
Comparison of CD4/CD8 ratio between the two timepoints.

**Figure 3:**
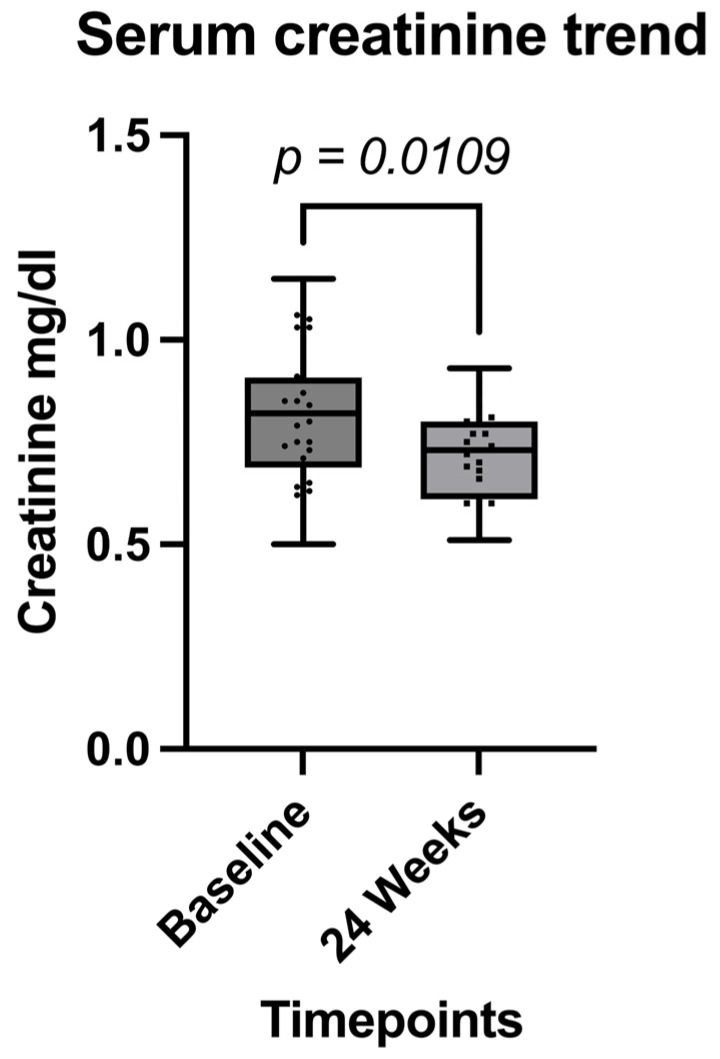
Comparison between serum creatinine levels between the two timepoints.

Immunological outcomes showed a statistically significant improvement in the CD4/CD8 ratio at 24 weeks (*p* = 0.0076) (Figure 2 and Table 5). Additionally, a significant reduction in serum creatinine levels was observed (*p* = 0.0109) (Figure 3), while no significant changes were detected in total cholesterol, triglycerides, alanine aminotransferase (ALT), or aspartate aminotransferase (AST) levels over the 24-week period (Table 2).

## 4. Discussion

This study contributes to the growing body of evidence supporting gender-specific research in HIV care by evaluating the virological and immunological outcomes of long-acting cabotegravir and rilpivirine (CAB/RPV LA) in women living with HIV (WLWH). While previous analyses from the SHiNe-SHiC cohort demonstrated the efficacy of oral bictegravir-based regimens in this population [13], our findings provide novel insights into the performance of injectable therapy in a real-world, gender-focused context.

At 24 weeks post-switch, the majority of participants maintained virological suppression, with an increase in the proportion achieving “target not detected” (TND) status. These results align with clinical trial data supporting the efficacy of CAB/RPV LA in diverse populations [17,18,19,20,21,22,23,24,25,26,27,28,29,30,31], but they are particularly relevant given the historical underrepresentation of women in such studies. Notably, our findings extend the evidence base by focusing exclusively on women, a group often overlooked in long-acting ART evaluations.

Immunologically, we observed a significant improvement in the CD4/CD8 ratio, a marker associated with immune recovery and reduced systemic inflammation. This early enhancement may reflect the sustained virological control achieved with CAB/RPV LA and suggests potential benefits beyond viral suppression. This critical improvement has been reported by Muccini et al. in a real-world setting in the general population [30]. Interestingly, a modest but statistically significant reduction in serum creatinine was also noted, contrasting with the mild increases typically reported with integrase inhibitors such as bictegravir and dolutegravir [13]. This observation warrants further investigation in larger cohorts.

Importantly, the safety profile of CAB/RPV LA was favorable, with only two discontinuations—one due to virological failure and one due to injection site reaction. No significant changes were observed in lipid or liver enzyme profiles, supporting the tolerability of this regimen in a population with frequent comorbidities, such as dyslipidemia and hypertension.

Despite its strengths, including a gender-specific focus and real-world applicability, this study has limitations. The retrospective design, absence of a comparator group, and relatively small sample size limit the generalizability of the findings. Nonetheless, this is, to our knowledge, one of the first real-world studies to assess CAB/RPV LA exclusively in women, highlighting the urgent need for more inclusive research in HIV treatment.

## 5. Conclusions

Our findings support the use of long-acting cabotegravir and rilpivirine (CAB/RPV LA) as a safe and effective treatment option for women living with HIV (WLWH), demonstrating high rates of virological suppression and early immunological improvement at 24 weeks post-switch. The observed increase in the CD4/CD8 ratio and the proportion of participants achieving “target not detected” status suggest meaningful clinical benefits in this underrepresented population.

This study adds to the limited real-world evidence on injectable ART in women and underscores the importance of gender-specific research in HIV care. While previous work from the SHiNe-SHiC cohort has highlighted the efficacy of oral regimens [13], our results provide complementary insights into the performance of long-acting therapy. Expanding access to such options may help address adherence challenges and improve long-term outcomes for women affected by HIV.

Further prospective studies with larger sample sizes and longer follow-ups are warranted to confirm these findings and explore the broader impact of CAB/RPV LA on quality of life, adherence, and comorbidity management in women.

## Figures and Tables

**Table 1:** Demographical and clinical characteristics of 54 women living with HIV switching to CAB/RPV.

Variable	Value
Age, median (IQR)	50 (36–57)
Ethnicity (n,%)	
Caucasian	50 (92.6%)
African	3 (5.6%)
Latin American	1 (1.8%)
Years since HIV onset (Median, IQR)	17.5 (6.75–29)
CD4 Nadir (Median, IQR)	258 (120–500)
Zenith VL (Median, IQR)	64,440 (8851–244,000)
Transmission Route (n, %)	
Sexual	49 (90.74%)
Injection drug user (IDU)	5 (9.26%)
Smokers (n,%)	19 (35.18%)
Alcohol use (n,%)	14 (25.93%)
Comorbidities (n,%)	
Hypertension	9 (16.36%)
Dyslipidemia	15 (27.27%)
Cancer	1 (1.81%)
Diabetes	3 (5.45%)
Anxiety	6 (10.91%)
Steatosis	2 (3.63%)
Previous Regimens (n,%)	
DTG-based 2DR	38 (70.37%)
DTG-based 3DR	3 (5.55%)
TAF-based	9 (16.67%)
Doravirine-based	4 (7.41%)

**Table 2:** Results of biochemical exams at baseline, 48 weeks, and 96 weeks in 54 females with HIV treated with CAB/RPV.

Parameter	Value at BL	Value at 24 w
WBC/mm^3^, Median (IQR),	6800 (5658–7775)	6900 (5543–7650)
Haemoglobin, g/dL, Mean (±SD)	13 (±1.2)	13 (±1.1)
Total cholesterol, mg/dL, Mean (±SD)	207 (±44)	194 (±35)
HDL Cholesterol, mg/dL, Mean (±SD)	55 (±16)	59 (±13)
LDL Cholesterol, mg/dL, Mean (±SD)	129 (±39)	115 (±35)
Triglycerides, mg/dL, Mean (±SD)	92 (±39)	99 (±54)

**Table 3:** Number of patients in different virological categories.

	TND	19–50	51–200	>200
BL	35	10	1	0
24 Weeks	39	4	2	1

**Table 4:** Contingency table of patients in virological categories between the two timepoints.

		24 Weeks			
		TND	19–50	51–200	>200
Baseline	**TND**	30	2	2	1
	**19–50**	8	2	0	0
	**51–200**	1	0	0	0

**Table 5:** Summary of main biochemical and immune–virological parameters at the two timepoints.

Parameter	Value at BL	Value at 24 w	*p* Value
Serum creatinine (mg/dL, mean, ±SD)	0.82 (±0.16)	0.72 (±0.12)	0.0109
CD4/mm^3^ (Median, IQR)	849 (641–1111)	850 (658–1138)	0.1914
CD8/mm^3^ (Median, IQR)	736 (534–928)	722 (528–863)	0.4217
CD4/CD8 Ratio (Mean, ±SD)	1.2 (±0.51)	1.3 (±0.59)	0.0076
Virological suppression, absolute count (VL < 50)	45 (97.8%)	43 (95.5%)	-

## Data Availability

The data presented in this study are available upon request from the corresponding author.

## Abbreviations

The following abbreviations are used in this manuscript:

WLWH	Women living with HIV
PLWH	People living with HIV
SHiNe-SHiC	Sardinian HIV Network and Sicilian HIV Cohort
ART	Antiretroviral therapy
CVF	Confirmed virologic failure
LA	Long-acting
ISR	Injection site reaction
TND	Target not detected
DTG	Dolutegravir
CAB/RPV	Cabotegravir/rilpivirine
TAF	Tenofovir alafenimide
BIC	Bictegravir
ALT	Alanine transaminase
AST	Aspartate transaminase
BL	Baseline
